# Similia Similibus Curantur

**Published:** 1881-07

**Authors:** Geo. F. Foote

**Affiliations:** Stamford, Conn.


### SIMILIA SIMILIBUS CURANTUR.

Geo. F. Foote, M. D., Stamford, Conn.

Paul was an innovator, writing his epistles some 1800 years ago; Galileo, at a later date, astonished the world with innovations upon the accepted physical sciences of his day; Harvey was an innovator, uttering new truths conflicting with the physiological teachings of the seventeenth century; Jenner, in 1785, offended the professional dignity of his confrères, by the innovation of a prophylactic nature, that has since been an accepted fact by nearly the whole world; Swedenborg, in advance of his age as a scientist, dared to give the world a new theology, illustrating the interior meaning of the Sacred Writings; about the same time, Hahnemann completely shocked the medical world, by promulgating a new law of cure.

These were all innovations upon established opinions made sacred by usage, while their authors met with coldness, persecution and martyrdom, patent to all who venture opinions and truths contrary to the mental platitudes that the popular will has been accepting as law.

We do not propose to worship these revealers of new truths. They were men like ourselves, but especially gifted for an end and an use. But we do choose to bend the knee, in humble adoration, to the Author of all good, for each and every new truth, that shall help the world to grow wiser and happier.

We look up to Hahnemann as a great man, especially prepared for a great work—a wise man. We regard him with veneration and with gratitude for his teachings, and for his courage in combatting the professional errors of his day, and for the substitution of a law in medicine that completely revolutionizes a practice so full of errors that we are simply astonished at its toleration and existence. *Similia similibus curantur* is deeply engraved upon the heart of every true Homœopathist. It expresses the great law of cure, before which every other supposed law sinks into insignificancy. If this is right, all others are wrong. That it is right, no one can doubt who has had experience in its application as the means of cure. If right, it is all right; in all cases and on all occasions.

The selected medicine under this law is not a "Base" that requires an "adjuvans;" the single remedy meets the occasion without a co-laborer. It calls for no car'rigen, because the medicine has no mischievous intent, and is fully able to do its work without any interference. It is not a triune made up of similar or opposing activities, but it is a triune with the simple but ever efficient law, that combines primaries, effects and ends. A principle its proceeding, and ultimate, a cause, a mode and uses. It is a true law, therefore a divine law. The world, not being prepared to accept the revelation of so great a truth, was a sufficient reason for its remaining in obscurity until the days of Hahnemann. Is comes to us even now, meeting an opposing force, made formidable through time, education and usage; a force, however, that is being weakened as the higher law reaches the intelligent observer of the present day. But it comes in a period of the world's history, that future generations will note as peculiarly marked for the development of many new truths, both moral and scientific, by which it is to be hoped the world is being made better; by which the sphere of man's usefulness is to be greatly enlarged, and his probationary existence gradually prolonged, until he attains the full measure of time allotted to an orderly life—which, in the estimation of the writer, will be an hundred and fifty years. And why not? If we but for a moment reflect upon the damaging effects of the vast amount of poisonous drugs that have for ages been poured into the human system, we cannot but arrive at the rational conclusion, that this perpetual disintegrading influence, from generation to generation, can have had no other effect than to degrade the organism and shorten the period of its existence; and then, if we need add to this, that other outbirth of this old heroic system of medical practice, viz.: the damning influence of stimulants, in their hydra-headed forms, as drinks, and as condiments with our food; also, the sedative and narcotizing influence of tobacco, all of which keep the system in a constant state of pyrexia, we are safe in the conclusion that the average of life is shortened by at least one-half; even with the few that attain to three score years and ten.

To those of us who have had much experience in this great law of similars, the thoughts of the future are pregnant with ambitious hopes. The practical application of this law when it becomes universal, is sure to prolong life; and that it will eventually become universal, the popularity with which it is fast being received, bears abundant testimony.

It is one of the great events of the present century. Its sublimity places it a discreet degree above the crucible test. Our limited knowledge of vital forces and of their receptivity and power, precludes any satisfactory explanations of its *modus operandi*. Therefore, we do not pretend to point out the line of communication or transit through the physical organization by which certain wellknown results are obtained. In consequence, we are content to accept for the present, at least, the simple knowledge of cause and effect, without the intermediate. Familiarity with results confirm us in opinions, against which antiquated and traditionary customs, can have no negative influences. We have passed the period of doubt, because of formidable innovations upon the earlier teachings. We have seen, therefore we know and believe; and as like causes produce like results, repeated experiences have confirmed us in a belief that is wholly irresistible, even against the sarcasm and ridicule that has been generously heaped upon us. We know whereof we talk, and guided by this mantle of truth, we are confident in our purpose, and are only sorry for those who have not had the opportunity for a belief that is so generous in its blessing to the suffering sick.

With this assurance, we will fill up the measure of our time, by a strict adherence to the laws of life, as seen from a rational standpoint; strictly adhering to the law of cure, that rational experimentation has taught us to be available, in every emergency where medicine is needed.

